# The Prognostic Significance of Anemia in Patients With Heart Failure: A Meta-Analysis of Studies From the Last Decade

**DOI:** 10.3389/fcvm.2021.632318

**Published:** 2021-05-13

**Authors:** Haijiang Xia, Hongfeng Shen, Wei Cha, Qiaoli Lu

**Affiliations:** ^1^Department of Cardiology, Affiliated Hospital of Shaoxing University, Shaoxing, China; ^2^Department of General Medicine, Zhuji People's Hospital of Zhejiang Province, Shaoxing, China

**Keywords:** anemia, hemoglobin, heart failure, mortality, hospitalization

## Abstract

**Background:** Anemia is a commonly occurring comorbidity in patients with heart failure (HF). Although there are a few reports of a higher prevalence of mortality and hospitalization-related outcomes due to accompanying anemia, other studies suggest that anemia does not have an adverse impact on the prognostic outcomes of HF. Two meta-analyses in the past decade had reported the adverse impact of anemia on both mortality and hospitalization- related outcomes. However, only one of these studies had evaluated the outcome while using multivariable adjusted hazard ratios. Moreover, several studies since then reported the prognostic influence of anemia in HF. In this present study, we evaluate the prognostic impact of anemia on mortality and hospitalization outcomes in patients with HF.

**Methods:** We carried out a systematic search of the academic literature in the scientific databases EMBASE, CENTRAL, Scopus, PubMed, Cochrane, ISI Web of Science, clinicaltrial.gov, and MEDLINE based on the PRISMA guidelines. Meta-analysis was then performed to evaluate the effect (presented as risk ratio) of anemia on the overall mortality and hospitalization outcome in patients with HF.

**Results:** Out of 1,397 studies, 11 eligible studies were included with a total of 53,502 (20,615 Female, 32,887 Male) HF patients (mean age: 71.6 ± 8.3-years, Hemoglobin: 11.9 ± 1.5 g/dL). Among them, 19,794 patients suffered from anemia (Hb: 10.5 ± 1.6), and 33,708 patients did not have anemia (Hb: 13.2 ± 1.7 g/dL). A meta-analysis revealed a high-odds ratio (OR) for the overall mortality in patients with anemia (OR: 1.43, 95% CI: 1.29–1.84). A high-risk ratio was also reported for hospitalization as the outcome in patients with anemia (1.22, 1.0–1.58).

**Conclusion:** This systematic review and meta-analysis provide evidence of the high risk of mortality and hospitalization-related outcomes in patients with HF and anemia. The study confirms the findings of previously published meta-analyses suggesting anemia as an important and independent risk factor delineating the prognostic outcome of chronic HF.

## Introduction

Heart failure (HF) is one of the most common types of cardiovascular disorders in the world (1, 2). According to the American Heart Association, HF is a complex cardiac syndrome that occurs as a result of a structural or functional dysfunction resulting in an impaired blood ejection across the body (3). Mentz and O'Connor (4) suggested a range of dysfunctions in the endothelial, renal system, and venous pathways that could eventually result in the remodeling of the myocardial structure leading to HF. Based on the statistics from the Global Burden of Disease Studies, HF is categorized as a rising global epidemic that accounts for more than 17 million deaths worldwide each year (5–7).

The patients with HF also exhibit a range of comorbidities that have a substantial impact on the prognostic outcome of the disease (8, 9), and the patient's quality of life (10). Studies have suggested that anemia is one of the most common comorbidities associated with HF (11–13). Recent studies have reported a high prevalence rate of anemia (28 to 58%) in patients with HF (14–16). The inception of anemia in patients with HF is largely associated with multifactorial reasons including deficits in iron metabolism, renal function, bone marrow function, and synthesis/response of erythropoietin (14, 15). Anand (17) suggested that the dysfunctional blunted response of erythropoietin due to the disruption in renal function as a result of HF could be one of the most critical factors contributing to the development of anemia. A substantial reduction in the renal flow of blood, in combination with dysfunctions in changes in the glomerular filtration rate, could influence the PO_2_ levels which ultimately has a detrimental effect on the structure of erythropoietin eventually leading to anemia.

Evidence also suggests that the adjunct medication-induced iron deficiency could explain the prevalence of anemia in patients with HF (18–20). Sirbu et al. (20) suggested that conventional drugs administered to patients with HF i.e., beta blockers, calcium channel blockers, Angiotensin converting enzyme inhibitor, and aspirin can substantially impair iron metabolism resulting in anemia.

In retrospect, anemia, accompanying HF, has an overall adverse impact on the prognostic outcome of patients. Nagatomo et al. (18) reported that anemia leads to an elevated hyper-hemodynamic in patients with HF. Moreover, increased cardiac workload by the means of stroke volume could also influence the sympathetic nervous activity which eventually can contribute to cardiac remodeling especially in the left ventricle, eventually promoting morbidity and mortality in HF patients (21, 22). Despite having an overall influence on the morbidity and mortality in the adult population, there is still no consensus regarding the prognostic influence of anemia on the mortality and hospitalization-related outcomes in patients with HF. Only a few high-quality studies have addressed the predominant impact of anemia on the prognostic outcome of HF patients in terms of mortality (23–26). However, only a few studies also report that anemia has no prognostic effect on the mortality and hospitalization-related outcomes in patients with HF (27, 28).

To date, only two systematic reviews and meta-analyses have reported the prognostic impact of anemia on the mortality and hospitalization-related outcomes in patients with HF (12, 29). Both of these studies reported a high relative risk ratio of mortality, hospitalization- related outcome in patients with HF, and anemia. However, several high-quality randomized controlled trials (23, 24), observation studies (25, 26), and retrospective cohort studies (30–32), that evaluate the prognostic influence of anemia on mortality and hospitalization outcomes in patients with HF, have been published recently. Therefore, there is a need for an updated systematic review and meta-analysis of the data.

This systematic review and meta-analysis provide an update on the current state of evidence regarding the prognostic influence of anemia in patients with HF, and risk ratios associated with the mortality and hospitalization-related outcomes in HF patients with anemia. The findings from the present study may provide cardiologists with a better understanding of the prognostic influence of anemia in patients with HF.

## Materials and Methods

A systematic review and meta-analysis was carried out based on the PRISMA (Preferred Reporting Items for Systematic Reviews and Meta-Analyses) guidelines (33).

### Data Search Strategy

Scientific databases (EMBASE, MEDLINE, CENTRAL, PubMed, Cochrane, ISI Web of Science, and clinicaltrial.gov, and Scopus) were searched from inception until September 2020. The following MeSH keywords: “heart failure,” “HF,” “Chronic heart failure,” “anemia,” “Hemoglobin,” and “Hb” were used in different combinations during the search across the academic databases. Manual screening of the bibliography section of the included studies were performed to identify further relevant studies. The inclusion criteria for the study were as follows:

- Studies that evaluated the prevalence of HF patients with anemia.
- Studies performed in the human population.
- Studies that evaluated the influence of anemia on the outcomes of short-, long-term mortality, and hospitalization outcomes.
- Studies published after 2008.
- Studies reporting the outcomes of mortality and hospitalization with the adjusted hazard ratio.
- Randomized-quasi-randomized and controlled clinical trials, observational prospective or retrospective studies.
- Studies published in peer-reviewed scientific journals or presented at conferences.
- English language studies.

The screening of the studies was independently performed by two reviewers. Cases of disagreements were resolved by discussions with a third independent reviewer. The following data were extracted from the included studies: author information, descriptive data, sample distribution, hemoglobin values, events of mortality, and events of hospitalizations. In cases of missing quantitative data, attempts were made to contact the corresponding authors of the publication.

### Quality Assessment

Quality (risk of bias) of each study was assessed by ROBINS-I, a Cochrane risk of bias assessment tool for RCTs randomized controlled trials and non-RCTs randomized controlled trials i.e., ROBINS-I (34, 35) and Cochrane risk of bias assessment tool for the randomized controlled trials (36). The ROBINS-I tool considers inadequate randomization, selective reporting, concealed allocation, classification, and missing data as major threats for instigating bias. The Cochrane tool for assessing randomized controlled trials assesses biases such as concealment of allocation, generation of random sequence, selective reporting, and blinding of outcome. The evaluation of methodological quality was done independently by two reviewers. Cases of disagreements were resolved by discussion with the third reviewer.

### Data Analysis

Meta-analysis of the included studies was done using the Comprehensive Meta-analysis software version 2.0 (37). The within group meta-analysis was performed using a random effects model (38). We estimated the pooled odds ratio from the included studies. Heterogeneity among the studies was assessed by *I*^2^ statistics, with the threshold for interpreting heterogeneity as follows: *I*^2^ statistics between 0 and 25%, negligible heterogeneity; 25–75%, moderate heterogeneity; and ≥75%, substantial heterogeneity (39). We distributed the data and performed the analysis for the overall mortality and hospitalization outcome, reporting odds ratio, confidence intervals (CI) of 95% level of significance, and heterogeneity. We also carried out separated sub-group analyses to evaluate the comparative influence of mortality between HF patients with a reduced and preserved ejection fraction. Publication bias was estimated using the Duval and Tweedy's trim and fill procedure (40). This non-parametric method tests for publication bias and adjusts the estimated overall effect size. Briefly an iterative method is used in which some of the extreme values remaining are removed, and a new mean effect is calculated. The alpha level of significance was set at 95%.

## Results

**Figure 1:**
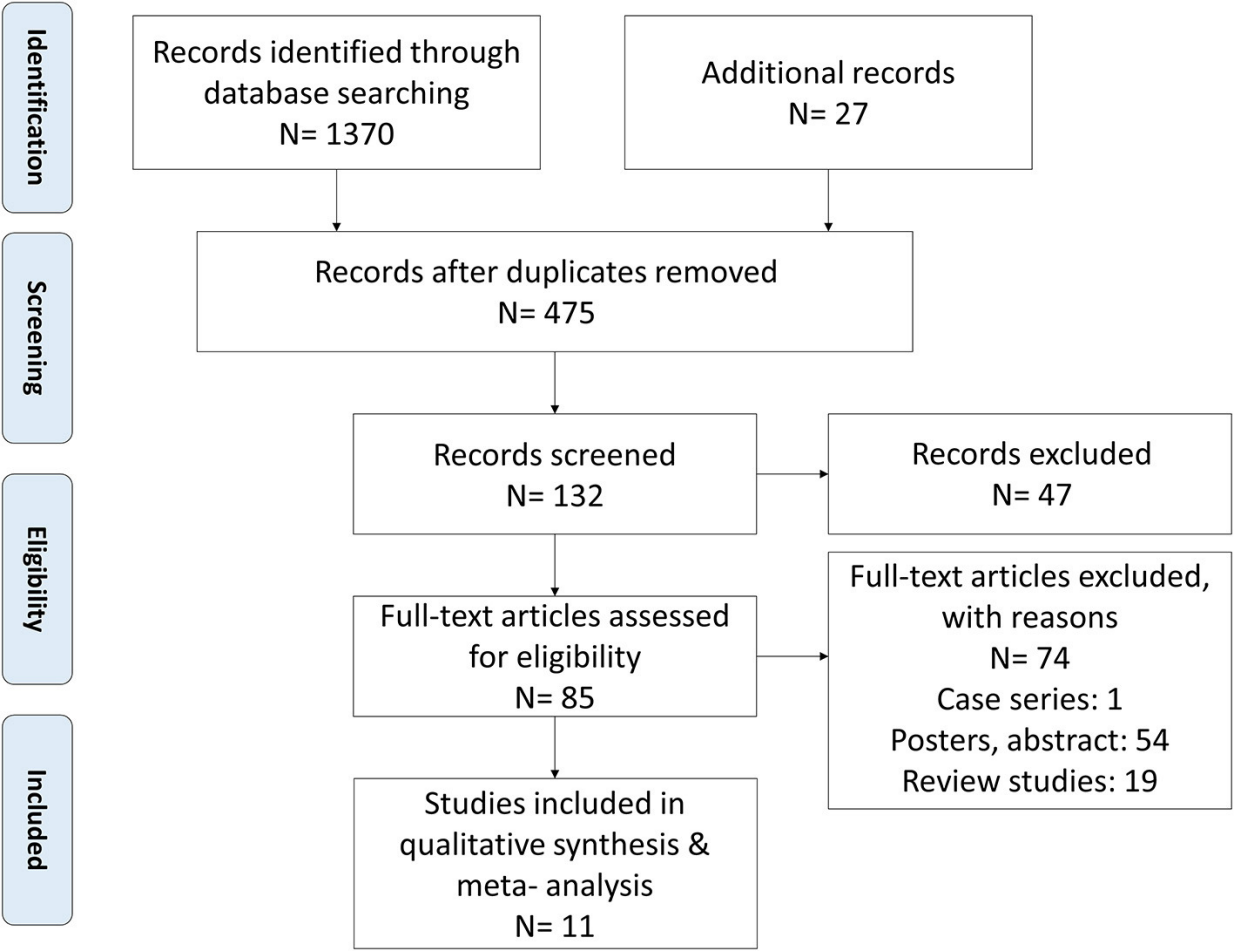
The PRISMA flowchart.

A systematic search resulted in 1,370 studies. Additional 27 studies were identified after screening the bibliography of articles (Figure 1). A total of 11 studies fulfilled the inclusion criteria. Three of the included studies were retrospective cohort studies (30–32), three were prospective cohort studies (41–43), two were randomized controlled trials (23, 24), two were observation studies (25, 26), and one was a retrospective observational study (44). The characteristics of the included studies are summarized in Table 1 and the clinical features of the patients are summarized in Table 2.

**Table 1:** Details of the included studies.

**Study**	**Country**	**Study design**	**Sample size (female, male)**	**Overall age (M ± SD) years**	**Mean overall Hb (g/dl) (Mean ± SD)**	**Patients sample distribution size (female, male)**	**Distributed sample age (Mean ± SD) years**	**Mean Hemoglobin (g/dl) (Mean ± SD)**	**Mortality (*n*)**	**Hospitalization (*n*)**
Ye et al. (32)	China	Retrospective cohort	3,279 (1,539 F, 1,740 M)	71	12.6	Anemia: 1,490 (742 F, 748 M) No anemia: 1,789 (797 F, 992 M)	Anemia: 74 No anemia: 67	Anemia: 10.7 No anemia: 14.1	Overall: 122 Anemia: 69 No anemia: 53	Overall: 1,817 Anemia: 801 No anemia: 1,016
Chairat et al. (30)	Thailand	Retrospective cohort	414 (228 F, 186 M)	62.6 ± 14.7	Male: 11.9 Female: 10.7	Anemia: 259 (153 F, 106 M) No anemia: 155 (75 F, 80 M)	Anemia: 64 No anemia: 58	Anemia: 10.0 No anemia: 13.6	Anemia: 52 No anemia: 19	Anemia: 79 No anemia: 47
Savarese et al. (25)	Sweden	Observational study	42,985 (15,926 F, 27,059 M)	75	12.7	Anemia: 14,779 (4,957 F, 9,822 M) No anemia: 28,206 (10,969 F, 17,237 M)	Anemia: 77 No anemia: 73	Anemia: 11.5 No anemia: 13.9	Anemia: 23,711 No anemia: 16,865	Anemia: 4,209 No anemia: 8,933
Gupta et al. (23)	USA	Randomized controlled trial	1,748 (872 F, 876 M)	72	12.8	Anemia: 716 (326 F, 390 M) No anemia: 1,032 (546 F, 486 M)	Anemia: 73 No anemia: 72	Anemia: 11.5 No anemia: 13.8	Anemia: – No anemia: –	Anemia: 451 No anemia: 516
Parcha et al. (24)	USA	Randomized controlled trial	215 (104 F, 111 M)	69	12.5	Anemia: 76 (27 F, 49 M) No anemia: 139 (77 F, 62 M)	Anemia: 71 No anemia: 67	Anemia: 11.5 No anemia: 13.5	Anemia: – No anemia: –	Anemia: 36 No anemia: 42
Kim et al. (44)	Korea	Retrospective observation study	384 (191 F, 193 M)	66.8	12.9	Anemia: 270 (139 F, 131 M) No anemia: 114 (52 F, 62 M)	Anemia: 71.3 ± 11.8 No anemia: 62.4±	Anemia: 11.3 ± 1.8 No anemia: 14.5 ± 1.5	Anemia: 48 No anemia: 12	Anemia: – No anemia: –
Jin et al. (42)	China	Prospective cohort study	1,604 (752 F, 852 M)	74.3 ± 11.3	12.4 ± 2.0	Anemia: 818 (393 F, 425 M) No anemia: 786 (359 F, 427 M)	Anemia: 76.9 ± 10.2 No anemia: 71.6 ± 11.8	Anemia: 10.9 ± 1.4 No anemia: 13.9 ± 1.3	Anemia: 143 No anemia: 83	Anemia: 367 No anemia: 296
Wienbergen et al. (26)	Germany	Observational study	949 (240 F, 709 M)	69.5	13.0	Anemia: 409 No anemia: 540	Anemia: – No anemia: –	Anemia: – No anemia: –	Anemia: – No anemia: –	Anemia: – No anemia: –
Formiga et al. (31)	Spain	Retrospective cohort study	155 (119 F, 36 M)	92.4 ± 2	11.7 ± 2.0	Anemia: 127 No anemia: 28	Anemia: – No anemia: –	Anemia: – No anemia: –	Anemia: 7 No anemia: 2	Anemia: 80 No anemia: 8
van den Berge et al. (43)	Netherlands	Prospective cohort study	1,769 (644 F, 1,125 M)	63.6	7.8	Anemia: 850 (285 F, 565 M) No anemia: 919 (359 F, 560 M)	Anemia: 63.1 ± 14.5 No anemia: 64.1 ± 15.0	Anemia: 6.7 ± 0.9 No anemia: 9.0 ± 0.8	Anemia: 365 No anemia: 257	Anemia: – No anemia: –
Goh et al. (41)	Singapore	Prospective cohort study	3,884 (827 F, 3,057 M)	60 ± 13	13.1 ± 2.1	Anemia: 1,606 (371 F, 1,235 M) No anemia: 2,278 (456 F, 1,822 M)	Anemia: 64 ± 13 No anemia: 58 ± 13	Anemia: 11.1 ± 1.2 No anemia: 14.5 ± 1.3	Anemia: – No anemia: –	Anemia: – No anemia: –

*M, Male; F, Female; n, number; SD, Standard deviation*.

**Table 2:** Clinical characteristics of the patients.

**Study**	**Cause of anemia**	**Diabetes (%)**	**Hypertension (%)**	**Chronic kidney disease (%)**	**Ejection fraction (%)**
Ye et al. (32)	Nd	A: 25 NA: 36.2	A: 39.1 NA: 45.6	A: 26.4 NA: 7.2	A: 50 NA: 40
Chairat et al. (30)	Nd	A: 31 NA: 15	A: 55 NA: 41	A: 24 NA: 1	Preserved: A: 64.2 NA: 62.2 Reduced: A: 29.6 NA: 28.3
Savarese et al. (25)	Nd	A: 33.1 NA: 24.9	A: 71.9 NA: 70.1	A: 50.3 NA: 43.1	–
Gupta et al. (23)	Nd	A: 57.1 NA: 36	A: 90.6 NA: 89.5	–	A: 51 NA: 51
Parcha et al. (24)	Nd	A: 39 NA: 53	A: 68 NA: 114	–	–
Kim et al. (44)	Nd	A: 44.8 NA: 18.4	A: 59.6 NA: 54.4	A: 9.6 NA: 1.8	–
Jin et al. (42)	Nd	A: 29.5 NA: 25.2	A: 74.7 NA: 72.9	A: 45.5 NA: 24.2	A: 61.6 NA: 61.4
Wienbergen et al. (26)	Iron deficiency	A: 37.6 NA: 35.1	–	A: 54.7 NA: 52.9	–
Formiga et al. (31)	Nd	–	–	–	–
van den Berge et al. (43)	Nd	A: 23 NA: 20	A: 32 NA: 34	–	–
Goh et al. (41)	Nd	A: 53 NA: 34	A: 59 NA: 51	A: 58 NA: 36	A: 29 NA: 27

*Nd, Not defined; A, Anemic; NA, Non-anemic*.

### Participant Information

A total of 57,386 patients with HF were included in the 11 studies, among them, 20,615 were females and 35,944 were males. In the included studies, three studies defined that their patients suffered from acute HF (31, 32, 44) and one had reported that they included cases with both acute and chronic HF (26). The rest of the seven studies did not provide details concerning the nature of the HF cases they included (23–26, 30, 41, 42). Besides, five of the included studies had also reported the ejection fraction values for their sample (23, 30, 32, 41, 42). The average ejection fraction for all the included studies was found to be 47.5 ± 15.2% for the HF patients with anemia, and 44.9 ± 15.6% for the HF patients without anemia. Moreover, the HF was defined by seven of the included studies with echocardiography (23, 24, 30, 32, 41, 42, 44), one study used the NT-proB-type Natriuretic Peptide blood test (25), and three studies did not define their measures for identifying HF (26, 31, 43).

Furthermore, the average age of the patients was 70.5 ± 8.6-years. All the included 11 studies had followed the World Health Organization classification to define anemia i.e., hemoglobin levels <13.0 g/dl for males and <12.0 g/dl for females. Moreover, among the anemic patients, mild anemia was defined as Hb ≥ 9.1 g/dl, moderate anemia was defined as 6.1 g/dl ≤ Hb <9 g/dl, and severe anemia was defined as Hb <6 g/dl. In our study, the average hemoglobin level was 12 ± 1.5 g/dL. In the subgroup distribution for the patients with/without anemia, a total of 21,400 patients have anemia, whereas 35,986 patients do not have anemia. Two studies did not report the gender distribution for this sub-sample of the patients with/without anemia (26, 31). From the studies that reported the gender distribution, a total of 2,931 females and 17,933 males have anemia, whereas 13,690 females and 21,728 males do not have anemia. The average age of the sub sample with and without anemia was 70.4 ± 5.4 and 65.9 ± 5.7-years, respectively. The average hemoglobin levels in the sub sample with and without anemia was reported to be 10.5 ± 1.5 and 13.4 ± 1.6 g/dL, respectively.

### Publication Bias

**Figure 2:**
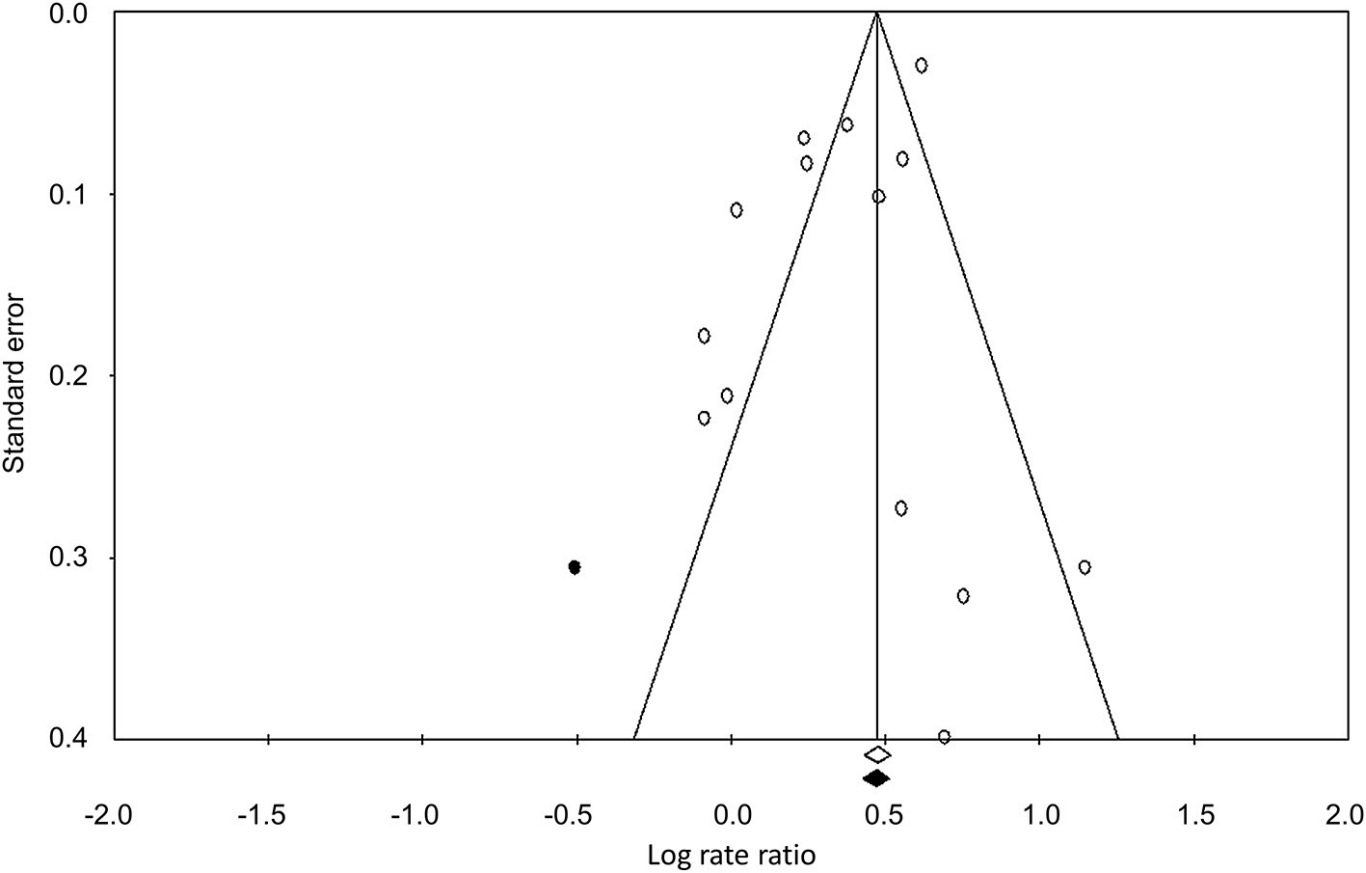
The publication bias by the Duval and Tweedy's trim and fill method.

The Duval and Tweedy's trim and fill method was used to identify any missing studies based on the random effect models on both sides of the funnel plot. The overall random effect models determined the point estimates and the 95% confidence intervals for all the combined studies as 1.42 (1.23–1.64). The results of the trim and fill method indicated that one study was missing on the left side of the funnel plot. The trim and fill method reported the imputed estimate of 1.37 (1.18–1.60). The publication bias is reported in Figure 2.

### Quality Assessment for Non-randomized Controlled Trials

**Figure 3:**
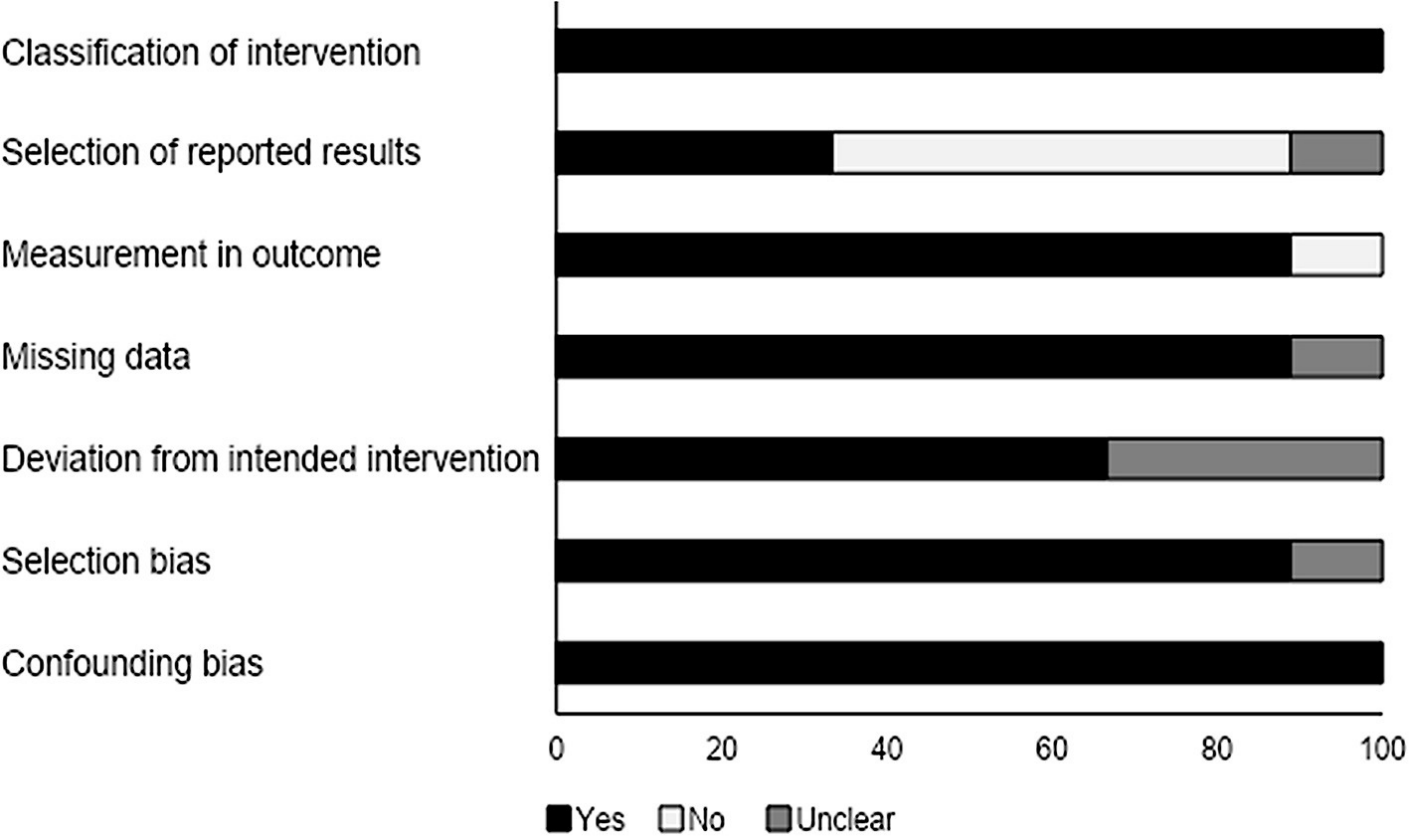
Risk of bias according to the Cochrane risk of bias assessment for the non-randomized controlled trials.

We analyzed the risk of bias in the methodology of the non-randomized controlled trials with the ROBINS-I tool (summarized in Table 3). The overall risk was found to be low in the included studies. We observed that the methodological risk of bias was highest for the selection of reported results and deviation from intended intervention. The overall risk of bias is summarized in Figure 3.

**Table 3:** Risk of bias within studies according to the ROBINS-I scale.

**Study**	**Confounding bias**	**Selection bias**	**Deviation from intended intervention**	**Missing data**	**Measurement in outcome**	**Selection of reported result**	**Classification of intervention**
Ye et al. (32)	+	+	+	+	+	+	+
Chairat et al. (30)	+	+	+	+	+	+	+
Savarese et al. (25)	+	+	+	+	+	+	+
Kim et al. (44)	+	+	+	+	+	–	+
Jin et al. (42)	+	?	?	+	+	?	+
Wienbergen et al. (26)	+	+	?	+	+	–	+
Formiga et al. (31)	+	+	?	+	+	–	+
van den Berge et al. (43)	+	+	+	+	+	–	+
Goh et al. (41)	+	+	+	?	–	–	+

### Quality Assessment for Randomized Controlled Trials

**Figure 4:**
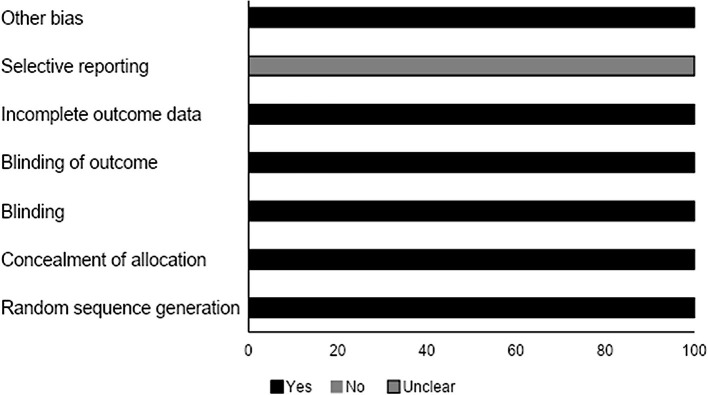
Risk of bias according to the Cochrane risk of bias assessment for the randomized controlled trials.

We analyzed the risk of bias for the randomized controlled studies using the Cochrane risk of bias assessment tool. Results are summarized in Table 4. The overall risk was low in the included studies. We observed an unclear risk of bias for the selective reporting section (Figure 4).

**Table 4:** Risk of bias within studies according to the Cochrane risk of bias assessment tool for randomized controlled trials.

**Study**	**Random sequence generation**	**Concealment of allocation**	**Blinding**	**Blinding of outcome**	**Incomplete outcome data**	**Selective reporting**	**Other bias**
Gupta et al. (23)	+	+	+	+	+	?	+
Parcha et al. (24)	+	+	+	+	+	?	+

### Meta-Analysis Report

#### Mortality Outcome

**Figure 5:**
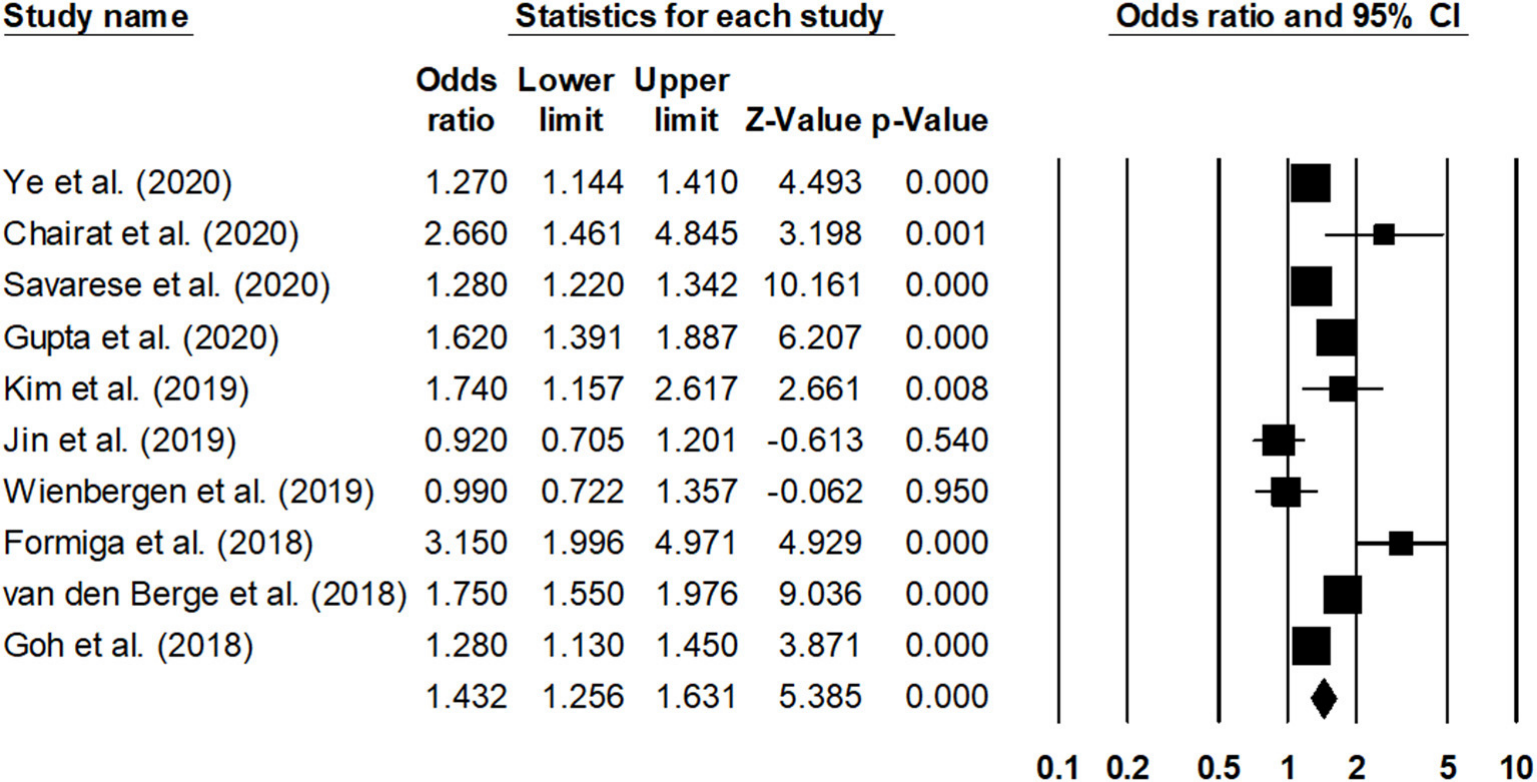
Forest plot for studies evaluating the overall 1-year overall mortality outcome. The adjusted odds ratios are presented as black boxes, whereas 95% confidence intervals are presented as whiskers. A negative odds ratio represents a reduction in the risk of mortality in heart failure patients with anemia, whereas the positive odds ratio represents an increase in the risk of mortality in heart failure patients with anemia.

The overall mortality rate was reported by 10 studies (25, 26, 31, 41–47). The outcome was reported by all the studies for a 1-year duration. The estimated pooled odds ratio was 1.43 (95% CI: 1.25–1.63, *p* < 0.001) (Figure 5), with moderate heterogeneity (*I*^2^: 56.1%).

**Figure 6:**
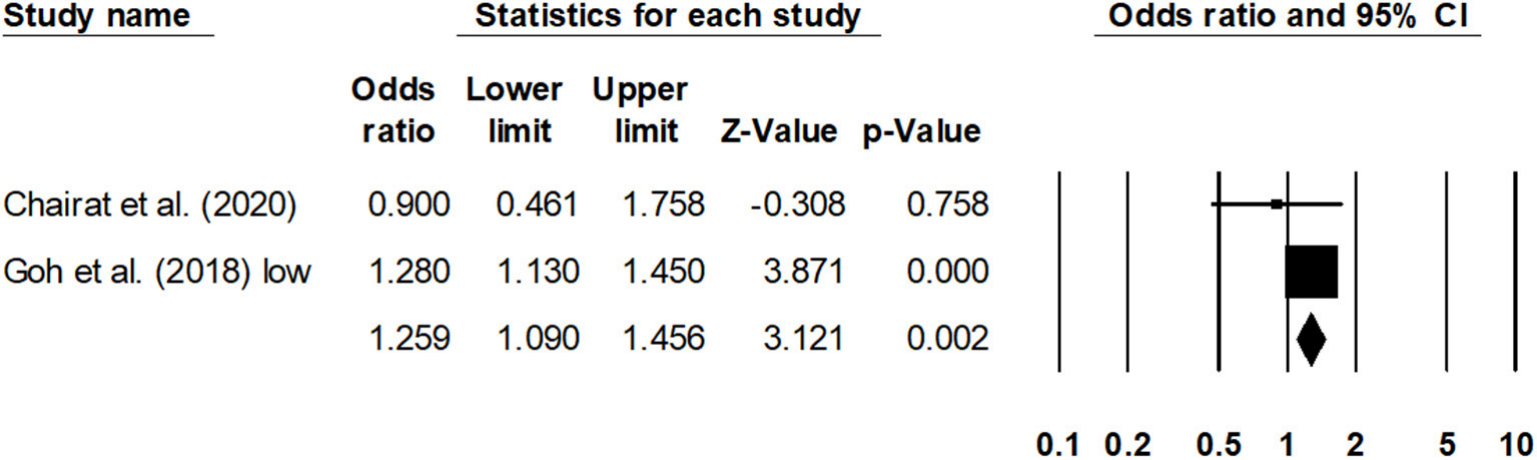
Forest plot for studies evaluating the overall 1-year overall mortality outcome for HF patients with reduced ejection fraction. The adjusted odds ratios are presented as black boxes, whereas 95% confidence intervals are presented as whiskers. A negative odds ratio represents a reduction in the risk of mortality in heart failure patients with anemia, whereas the positive odds ratio represents an increase in the risk of mortality in heart failure patients with anemia.

**Figure 7:**
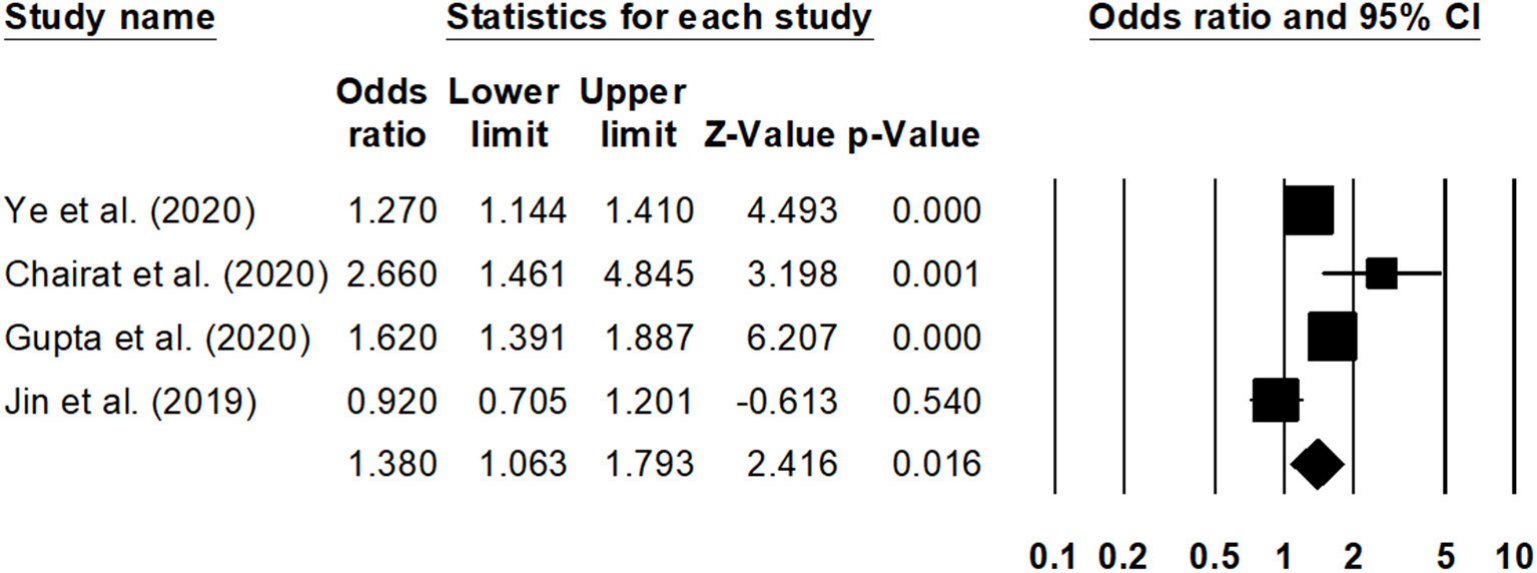
Forest plot for studies evaluating the overall 1-year overall mortality outcome for HF patients with preserved ejection fraction. The adjusted odds ratios are presented as black boxes, whereas 95% confidence intervals are presented as whiskers. A negative odds ratio represents a reduction in the risk of mortality in heart failure patients with anemia, whereas the positive odds ratio represents an increase in the risk of mortality in heart failure patients with anemia.

Sub-group analysis for the overall mortality rate with a reduced ejection fraction was reported by two studies (30, 41). The estimated pooled odds ratio was 1.25 (95% CI: 1.09–1.45, *p* < 0.01) (Figure 6), with no heterogeneity (*I*^2^: 0%). Likewise, sub-group analysis for overall mortality rate with a preserved ejection fraction was reported by four studies (23, 30, 32, 42). The estimated pooled odds ratio was 1.38 (95% CI: 1.06–1.79, *p* < 0.02) (Figure 7), with moderate heterogeneity (*I*^2^: 48.5%).

#### Hospitalization Outcome

**Figure 8:**
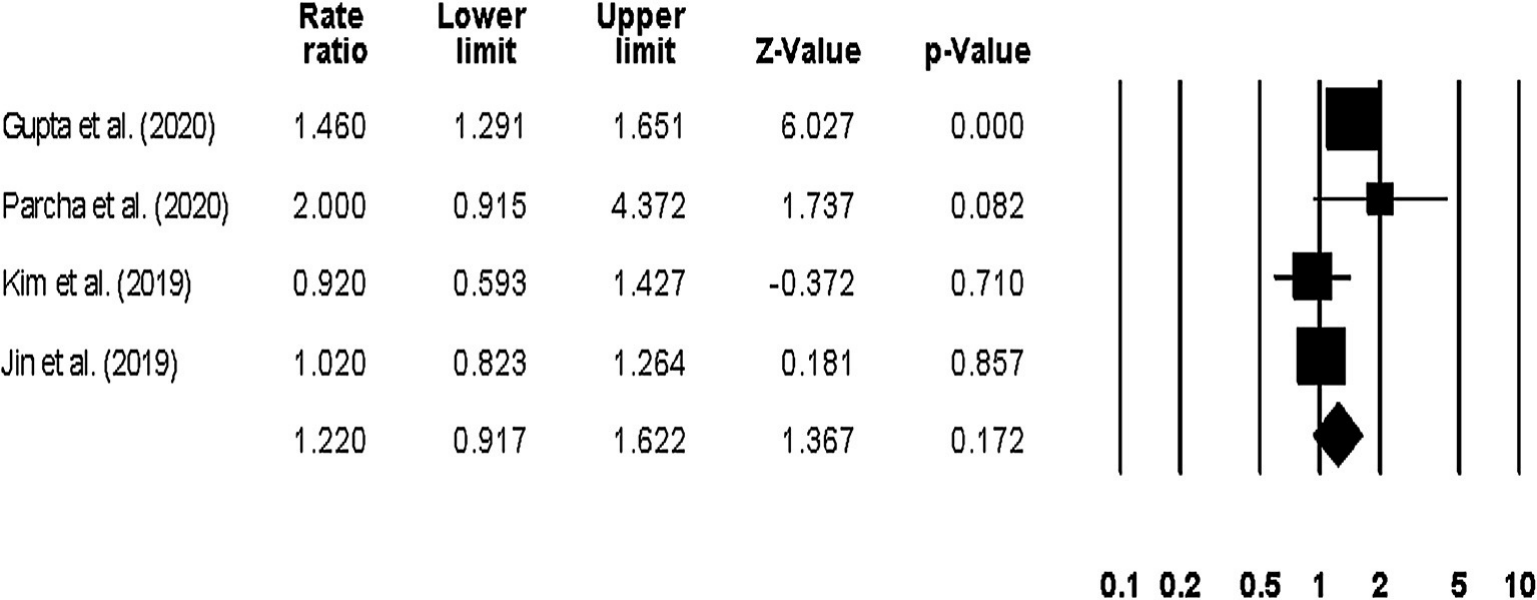
Forest plot for studies evaluating the overall hospitalization outcome. The adjusted odds ratios are presented as black boxes, whereas 95% confidence intervals are presented as whiskers. A negative odds ratio represents a reduction in the risk of mortality in heart failure patients with anemia, whereas the positive odds ratio represents an increase in the risk of mortality in heart failure patients with anemia.

The overall hospitalization outcome was reported by four studies (23, 24, 42, 44), with two studies reporting the outcome at a 1-year follow up (23, 42), one at a 1.5-year follow up (44), and one after 24-weeks (24). The pooled rate ratio was 1.22 (95% CI: 1.0–1.58, *p*: 0.04) (Figure 8), with no heterogeneity (*I*^2^: 0%).

**Figure 9:**
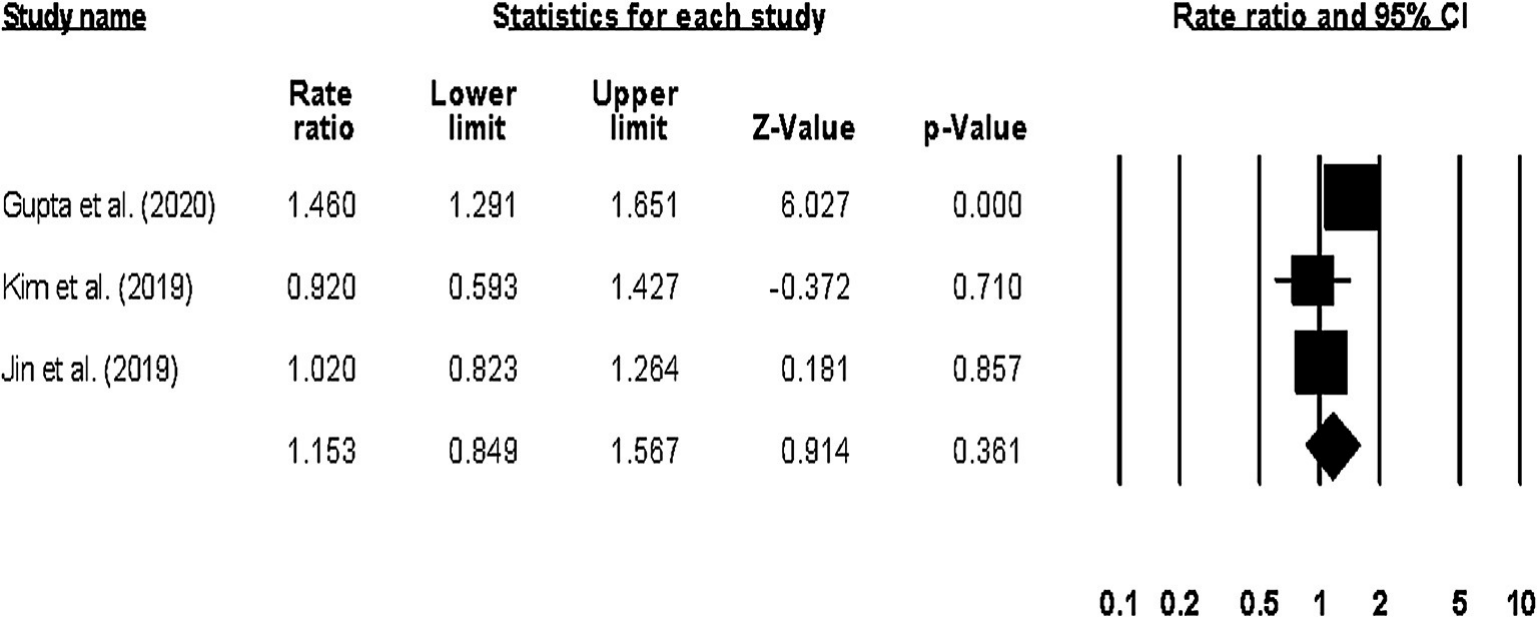
Forest plot for studies evaluating the overall long-term hospitalization outcome. The adjusted odds ratios are presented as black boxes whereas 95% confidence intervals are presented as whiskers. A negative odds ratio represents a reduction in the risk of mortality in heart failure patients with anemia, whereas the positive odds ratio represents an increase in the risk of mortality in heart failure patients with anemia.

Sub-group analysis on only long-term (1–1.5-years) hospitalization outcomes showed a pooled risk ratio of 1.15 (95% CI: 0.84–1.56, *p*: 0.36) (Figure 9), with no heterogeneity (*I*^2^: 0%).

## Discussion

This systematic review and meta-analysis provide a comprehensive state of evidence regarding the prognostic influence of anemia in patients with HF. We observed high overall risk ratios associated with the overall mortality and hospitalization-related outcomes in HF patients with anemia.

The management of HF is one of the most challenging aspects for cardiologists worldwide due to its atypical pathophysiological mechanism, coexisting morbidities, and manifestations (48–50). Presence of comorbidities, such as anemia, makes it even more challenging for clinicians to evaluate the cardiovascular condition of the patient, thus potentially contributing to an increased morbidity and mortality-related outcomes (12, 51, 52). Recent literature has increasingly recognized the rising prevalence of anemia in patients with HF (53–55). Several mechanisms are linked with the aggravation of anemia in patients with HF (14, 17–19). The study by Anand and Gupta (14) suggests that the absolute/functional nutritional deficiency of iron, response/synthesis of erythropoietin, and adverse effects of medications are the most significant factors aggravating the onset of anemia. Moreover, the increase in pro-inflammatory markers in patients with HF could also contribute to anemia by activating the GATA-binding proteins and nuclear factor kappa light chain enhancer, eventually inhibiting the production of renal erythropoietin or resulting in a blunted-erythropoietin response (56–58). In addition to these inflammatory changes, deficiency and/or mutation of genes that regulate hematopoiesis have also been reported to eventually worsen cardiac remodeling i.e., hypertrophy in the left ventricle (59, 60). These changes eventually promote the increased risks of morbidities that can lead to re-hospitalizations and eventually to an increased mortality (12).

In our present review, we analyzed a range of studies that reported a predominant influence of anemia on all-cause 1-year mortality related outcomes in patients with HF. Ye et al. (32) evaluated the overall all-cause mortality in 3,279 patients and reported a significant (*p*: 0.01) increase in the mortality-related events in HF patients with anemia as compared to patients without anemia. This relationship between anemia and mortality was proportional to the severity of the anemia i.e., the more severe the anemia, the higher the mortality rates. Similarly, Jin et al. (42) reported higher mortality related outcomes for the anemic group (14.6%) as compared to the non-anemic group (8.7%) in a Chinese cohort of 1,604 patients. The authors further showed that the mortality rates in anemic patients further increased in patients with renal dysfunction (21%). This increase in mortality-related events in HF patients with anemia may be due to several mechanisms, such as enhancement in oxidative stress, fluid-retention, cardiac-hypoxia, sympathetic nervous activity, and renin-angiotensin-aldosterone activity (61, 62). Sanderson (63) further suggested that a decrease in the neuro-hormonal activation as a result of a reduced vascular-resistance and decreased blood viscosity could further reduce the glomerular filtration rate and enhance the overall retention of water and salt content leading to an expanded extracellular plasma volume, eventually increasing the morbidity and mortality related outcomes in patients with HF. In our present meta-analysis, we confirm these findings and report a high-odds ratio (OR: 1.43, 95% CI: 1.25–1.63) associated with the overall mortality-related factors in patients with HF. Besides, in a further subgroup comparative analysis of mortality between HF patients with a reduced and preserved ejection fraction, we observed differences in terms of mortality between the two groups.

We also assessed the risk of hospitalization-related outcomes associated with anemia in patients with HF. We observed that all the studies included in our systematic review had reported an overall increase in the hospitalization-related outcomes for HF patients with anemia. Kim et al. (44) showed a high rate of re-hospitalization in anemic patients (Hazard ratio: 0.92, 0.59–1.42) as compared to non-anemic patients with HF. The authors also suggest that the anemia diagnosis at discharge could serve as a predictor not only for the morbidity-but also for the mortality-related outcomes, thus emphasizing the importance of managing anemia during hospitalization to reduce the adverse events in patients with HF. RELAX randomized controlled trial by Parcha et al. (24) reported a higher incidence of hospitalization in anemic patients primarily due to cardiac or renal problems even at the end of a 24-week period (24). The authors suggest that the timely recognition and management of anemia could be crucial for stemming the deterioration of the symptomatic manifestations in patients with HF.

Our meta-analysis is in agreement with these reports. We showed high risk ratios of re-hospitalizations in anemic patients with HF (1.22, 1.27–1.78). Subgroup analysis of long term (>1-year) hospitalization-related outcome also revealed a high-risk hospitalization ratio in anemic patients with HF (1.15, 0.84–1.56).

Our systematic review and meta-analysis have a few limitations. First and foremost, this systematic review and meta-analysis was not registered in a review repository such as PROSPERO. Although the lack of registration might raise concerns regarding the validity of this present review (64), we would like to assure our reader that we made attempts to register our review at these repositories, but because of the current pandemic crisis, the waiting time at the PROSPERO repository was >1-year. Secondly, we did not evaluate the gender differences in terms of the risks of hospitalization and mortality associated with anemia in patients with heart failure. We understand the importance of evaluating the gender differences in terms of the prognostic significance it possesses for clinicians. Therefore, we strongly recommend future studies to address this limitation by conducting gender sub-group analysis to outline the gender differences in terms of the prognostic outcome of anemia in HF. Thirdly, our understanding of the short- and long-term hospitalization related outcomes in HF patients with anemia may be biased due to an insufficient data in the eligible included studies. In our included pool of four studies that had reported the hospitalization related outcomes in HF patients with anemia, only one study had evaluated the outcome during a 24-week follow-up (24), whereas the other three studies had evaluated the long-term (1–1.5-years) outcomes (23, 42, 44). Therefore, the outcomes of risk ratio overall hospitalization could be biased, and we would suggest our reader to evaluate this statistic with caution. Additionally, short- and long-term prognostic influences of anemia on mortality were not evaluated in this review and meta-analysis, since all the included studies had reported a 1-year mortality outcome. Previous studies have stressed upon the high risks of mortality-related outcomes especially during the short-term periods (43). Therefore, future studies are needed to address these limitations by conducting more high-quality studies and sharing their descriptive data in open access data repositories. The evaluation of these outcomes would be highly beneficial for medical practitioners to predict the prognostic outcomes regarding mortality and re-hospitalization in HF patients with anemia. Lastly, we would like to mention that usually an important limitation in the literature involving patients with heart failure is that the researchers do not report the definitions of anemia they adopted. In this present study, however, we found that all the included studies had adopted the World Health Organization's classification to define anemia i.e., Hb < 13.0 g/dl for males and Hb < 12.0 g/dl for females.

In conclusion, in this present systematic review and meta-analysis, we provide a confirmatory evidence regarding the high prognostic influence of anemia in patients with HF. We provide statistical evidence of the high mortality and hospitalization-related risks associated with anemia in patients with HF. The findings from the present study can further contribute in developing clinical awareness of the widespread prevalence of anemia in patients with chronic HF. This may help cardiologists to develop the best practice guidelines for determining the appropriate treatment approach for controlling adverse outcomes in HF patients with anemia.

## Data Availability Statement

The original contributions presented in the study are included in the article/supplementary files, further inquiries can be directed to the corresponding author/s.

## Author Contributions

HX conceived and designed the study and was involved in the writing of the manuscript. HS, WC, and QL collected the data and performed the literature search. All authors have read and approved the final manuscript.

## Conflict of Interest

The authors declare that the research was conducted in the absence of any commercial or financial relationships that could be construed as a potential conflict of interest.
